# A seismic metamaterial: The resonant metawedge

**DOI:** 10.1038/srep27717

**Published:** 2016-06-10

**Authors:** Andrea Colombi, Daniel Colquitt, Philippe Roux, Sebastien Guenneau, Richard V. Craster

**Affiliations:** 1 Dept. of Mathematics, Imperial College London, South Kensington Campus, London, UK; 2 Dept. of Mathematical Sciences, University of Liverpool, UK; 3 ISTerre, CNRS, Univ. Grenoble Alpes, Grenoble, France; 4 Institut Fresnel-CNRS (UMR 7249), Aix-Marseille Université, Marseille, France

## Abstract

Critical concepts from three different fields, elasticity, plasmonics and metamaterials, are brought together to design a metasurface at the geophysical scale, the resonant metawedge, to control seismic Rayleigh waves. Made of spatially graded vertical subwavelength resonators on an elastic substrate, the metawedge can either mode convert incident surface Rayleigh waves into bulk elastic shear waves or reflect the Rayleigh waves creating a “seismic rainbow” effect analogous to the optical rainbow for electromagnetic metasurfaces. Time-domain spectral element simulations demonstrate the broadband efficacy of the metawedge in mode conversion while an analytical model is developed to accurately describe and predict the seismic rainbow effect; allowing the metawedge to be designed without the need for extensive parametric studies and simulations. The efficiency of the resonant metawedge shows that large-scale mechanical metamaterials are feasible, will have application, and that the time is ripe for considering many optical devices in the seismic and geophysical context.

What if in the future the propagation of seismic surface waves around critical infrastructure could be shaped at will? Until a few years ago, this question would have sounded provoking to the seismological community. Today, thanks to seismic metamaterials, this is no longer the case. The use of metamaterials to control the way in which waves travel was popularised by Pendry *et al*.^1^ and Leonhardt^2^, who introduced the concept of transformation optics in 2006. Transformation optics can be understood in terms of Faraday’s picture of electric and magnetic fields as lines of force^3^; these lines of force can be manipulated, almost at will, via carefully designed effective material properties (tensors of permittivity and permeability). Similarly, This concept can be applied to elastic waves from the laboratory scale^4^,^5^ up to the geophysical scale^6^,^7^ despite underlying theory, governing equations, and physics of mechanical and seismic waves are fundamentally different to those in electromagnetism. The exploration of the analogies between the electromagnetic and elastic metamaterial realms keeps delivering surprising new phenomena as the one presented in this study. By applying concepts derived from photonics to a recently developed metamaterial for seismic Rayleigh waves^7^, we translate the optical rainbow effect^8^ into a unique seismological effect, and show that it is possible to construct a metasurface capable of controlling the flow of Rayleigh waves. A seismic Rayleigh wave is a mechanical wave that travels over the surface of the Earth and is partially responsible for far-field damages associated with earthquakes^9^. The control of this type of waves is an essential paradigm for seismic protection of structures via metamaterials.

A key concept of the optical rainbow effect of Tsakmakidis *et al*.^8^ is the graded wedge of subwavelength resonators that can trap and spatially segregate (as a rainbow) the different frequencies (colours) of light. This seminal work has naturally generated substantial interest in the photonics and plasmonics communities with researchers subsequently showing that the optical properties of surface plasmon polaritons (SPPs)^10^ can be tailored, by varying the surface nanotopology, via so-called Surface Dispersion Engineering^11^,^12^,^13^,^14^,^15^,^16^. Furthermore, similar ideas are finding application outside of plasmonics with the recent demonstration of the acoustic analogue of the optical rainbow^17^,^18^,^19^. At a much bigger scale, a forest densely populated with trees represents a *naturally occurring* geophysical metamaterial for Rayleigh waves. Forests of trees have been first modelled, in a laboratory environment, using thin rods attached to a metallic plate^5^,^20^ and conclusively validated with an experiment in an actual forest environment by Colombi *et al*. Trees can be easily approximated by elastic rods attached to a bulk elastic substrate. The resonances of the rods in the plate, or of the trees, in the case of the forest, play the role of the electric currents in the optical rainbow^8^ creating effective material properties (stiffness tensors) that allow for the control of seismic surface waves.

## Results

Here we unite critical concepts from several seemingly disparate fields including, plasmonics, metamaterials, elasticity, and geophysics to design a *seismic metawedge* capable of creating a *seismic rainbow* to mode convert the destructive Rayleigh seismic waves into harmless bulk shear waves. It is important to emphasise that optics and acoustics do not posses a polarisation analogous to the Rayleigh wave; in particular, the recent experiments with acoustic waves in graded grooved waveguides^17^,^18^ do not support mode conversion and can only demonstrate the classical acoustic rainbow effect.

**Figure 1:**
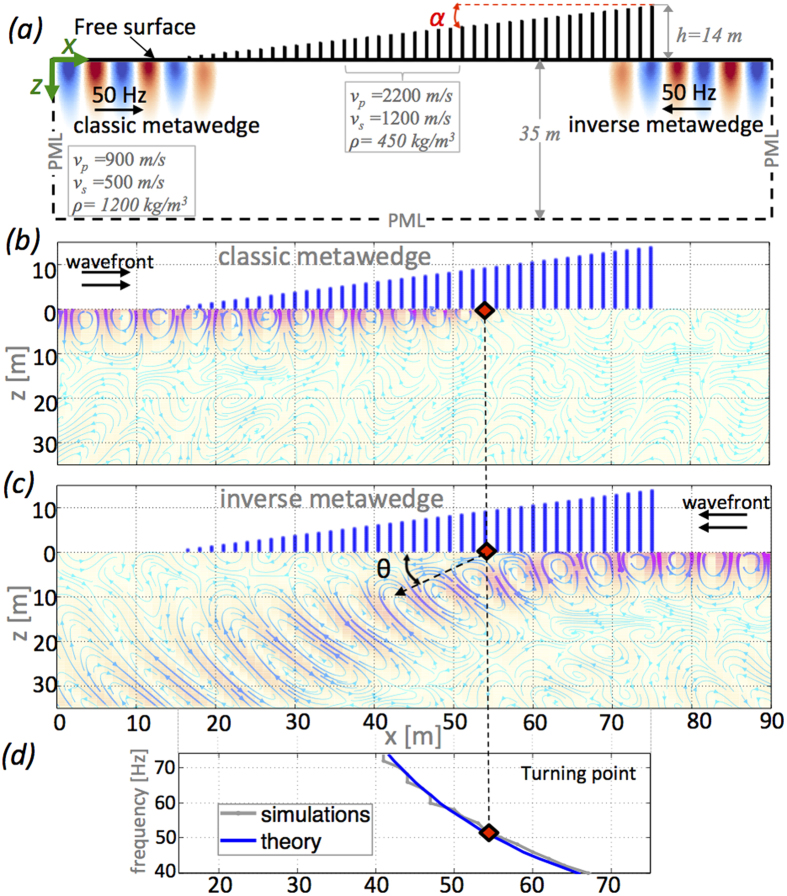
The metawedge of angle 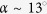
showing, in (**a**), the geometry and material parameters. In (**b**,**c**) the elastic streamlines where the colorscale and linewidth represent the magnitude of the displacement field; the red square indicates the turning point from Eq. 1. Panels (b,c) are for increasing, and decreasing, respectively resonator height relative to the direction of Rayleigh wave incidence. Perfectly matched layers (PML) mimic unboundedness in the numerical simulations. Panel (d) gives the theoretical prediction (Eq. 1) versus measured, from numerical simulation, of the turning point position for various frequencies for the wedge in (**a**).

To succinctly illustrate the concepts behind the resonant metawedge as a geophysical metamaterial, we model a graded array of vertical resonators in 2D (Fig. 1) using the methodology of^7^. The coupling between Rayleigh waves and the longitudinal resonances of the vertical resonators, which is dependent upon the seismic impedance match between substrate and metawedge, creates large bandgaps bounded below by frequencies inversely proportional to the resonator height *h*^7^; only the first longitudinal mode, and hence the first bandgap, is relevant for the design of the metawedge. This first resonance frequency is:


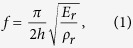


where the resonator is characterised by, *h*, its height, *E*_*r*_, Young modulus and *ρ*_*r*_ its density. The resonant metawedge is quickly obtained from a graded array of vertical resonators (Fig. 1a) as *f* is simply tuned by adjusting the height *h*. Previous studies have proven that a 2D model is perfectly suited to analyze the physics of this metamaterial and this simplification does not affect the validity of the results in 3D^7^,^21^. Conversely, it allows quantitative analysis of the seismic rainbow phenomenon and a rapid evaluation of its sensitivity to model parameters. Furthermore, the 2D symmetry of the model in Fig. 1 limits the type of resonant modes of the 2D resonators to the longitudinal and flexural ones, the same that characterise slender vertical resonators with cylindrical cross section in the 3D case. The flexural modes contribute very marginally to the dispersion properties of this metamaterial^7^, and therefore, they will not be further discussed.

The illustrative metawedge in Fig. 1a is designed as an array of 40 resonators whose height smoothly increases from 1 to 14 m (in terms of area this is roughly 5 resonators per wavelength). Given this height-range, the resonance frequencies and range of protected frequencies, span approximately a 30–120 Hz frequency band (from Eq. 1). Because we are dealing with a locally resonant metamaterial^7^, resonators in the wedge can be randomly spaced. Here we make use of equal spacing to simplify the design and ease the interpretation of the results. Therefore resonators are spaced 2 m apart, the resulting metawedge angle *α* is 13°, and the mechanical properties used are given in Fig. 1a; this is simulated numerically using the time evolution spectral element code SPECFEM2D^22^. In Fig. 1a–c the input signal is made of monochromatic Rayleigh waves at 50 Hz and we show two cases with Rayleigh waves incident towards the shorter, or taller, end of the metawedge.

**Figure 2:**
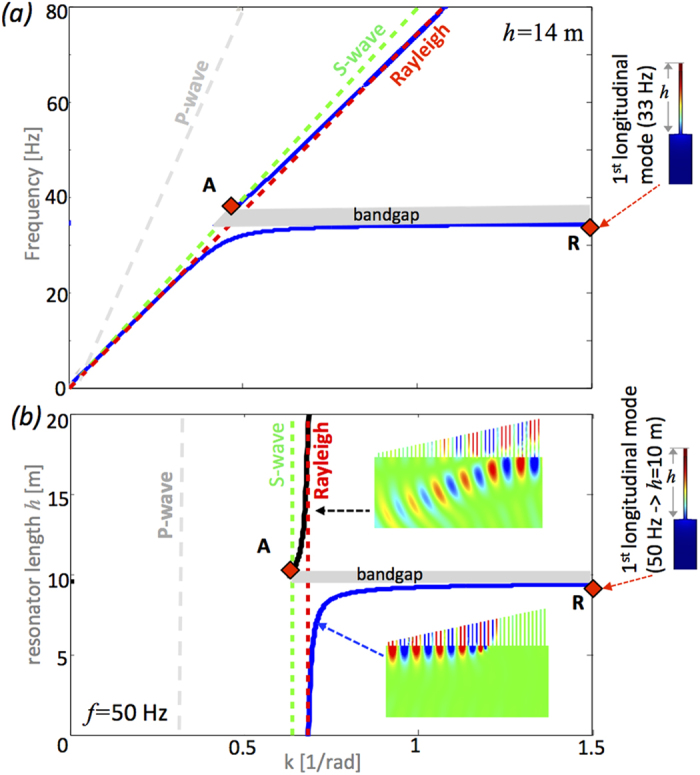
Dispersion curves. In panel (a) the conventional view of frequency versus Bloch wavenumber for a vertical resonator of fixed height *l* = 14 m. Note the points *A*, where S-wave and Rayleigh wave hybridise, and *R*, the resonance frequency, that bound the band-gap. Panel (b) shows the variation of resonator height with Bloch wavenumber at fixed frequency which shows the change in wave character associated with the different dispersion curves.

When the waves are incident on the short edge we obtain the classic wedge (Fig. 1b) analogous to the acoustic rainbow^17^ with the wave trapped. Conversely when incident on the taller end we observe Rayleigh to shear wave conversion (Fig. 1c). The motion polarization inside the substrate is well captured in Fig. 1b,c where the streamlines are plotted together with field magnitude particularly notable are the elliptical streamlines characterising the Rayleigh waves propagating along the free surface. To aid interpretation of these numerical simulations, we have derived the analytical form of the dispersion curves (full details given in the supplementary material); these are plotted in Fig. 2a for a regular array of 14 m constant height resonators. For a substrate without resonators the dispersion curves are just those of bulk (P and S) and surface Rayleigh modes, the addition of the vertical resonators induces hybridization between the Rayleigh waves and the subwavelength resonators that introduces an hybrid mode (blue line) characterized by a bandgap and a Fano-like shape^5^,^23^. Points R and A demarcate the bandgap region. The point labelled R represents the longitudinal resonance (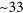 Hz) derived from Eq. 1 and A is given by the intersection of the hybrid mode (blue line) and the S-mode (green dashed line). This point A, and the hybridization, plays a key role in understanding the inverse wedge of Fig. 1c. The frequency at A is obtained from the dispersion relationship (see the supplementary material) and for the fundamental mode it is:


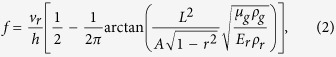


where *v*_*r*_ is the the compressional wave-speed in the resonator, *μ*_*g*_ and *ρ*_*g*_ are the shear modulus and density of the substrate respectively, *A* and *h* are the cross-sectional area and height of the resonators respectively, *L* is the lattice constant of the array of resonators, and *r* is the ratio between shear and compressional velocity *v*_*s*_/*v*_*p*_ in the substrate. In Fig. 2a the S-wave dispersion line splits the *f* − *k* plane into propagative and evanescent regimes, respectively, located to the right and to the left of the line. Flexural modes, although present in the numerical simulations, play a minor role and they are not represented^7^. As the resonator height varies across the wedge, the dispersion curves of Fig. 2a, derived for constant resonator height, offer a limited but nonetheless valuable view of the physics. Fig. 2b is an unconventional view of the dispersion, in the *h* − *k* plane at a fixed frequency of 50 Hz (i.e. the case in Fig. 1b,c), as we show that the height variation is critical for the observed effects and gives us a clear picture of the dispersion as waves travel through the wedge: At this frequency, resonance occurs for an approximately 10 m high resonator.

For the classic wedge case (Fig. 1b), the ‘rainbow’ effect, the incident wavefront approaches the wedge, from the left towards the short edge of the metawedge, initially undisturbed as a Rayleigh wave with characteristic elliptical polarization. After travelling a few wavelengths inside the wedge, it slows down until reaching the resonator whose fundamental longitudinal mode matches the input frequency of the signal (50 Hz in the figure). This position is dubbed the turning point because the wave trajectory is forced to diverge (red squares in Fig. 1b,c). At this stage the propagation is characterized by a bandgap which, like a rigid barrier, reflects the energy backward. This situation is interpreted using the blue branch of the dispersion curve in Fig. 2b; the wavefront initially encounters very short resonators and it propagates as a Rayleigh mode. As the resonators become taller (>5 m), the group velocity is progressively reduced until the bandgap is reached at point R. The turning point is easily predicted starting from Eq. (1) for different frequencies or wedge angles *α*. Figure 1d shows the *x*-coordinate of the turning point as a function of the input frequency and the theoretical predictions match perfectly with the numerical simulations. The existence of a turning point (or equivalently a bandgap) inside the wedge for all these frequencies means that this device, acting as an ultra broadband shield for Rayleigh waves, can be employed to fabricate geophysical metamaterials.

We now move to the intriguing case of the inverse wedge. In Fig. 1c the Rayleigh waves now approach the wedge from the right towards the taller end of the metawedge and the wavefield follows a very different pattern vis-a-vis the classic wedge. At the turning point the wavefront is mode-converted and directed into the interior of the substrate with the Rayleigh waves, characterized by *x* and *z*-components, converted into a S-wave whose motion is polarized in the transverse direction. Confirming this interpretation is geometrical spreading, typical of body waves, that can be clearly observed after the conversion. Using Fig. 2b to interpret the effect of the height variation we notice that the black branch of the dispersion curve initially follows the Rayleigh line and it is progressively steered towards the S-line. It is in this region, between Rayleigh and shear lines, that the conversion takes place. Whilst a bandgap still exists for the Rayleigh mode, as in the classic wedge, the elastic energy avoids the reflection by conversion to S-waves that propagate into the substrate. This converted wave propagates at a refraction angle of 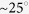 calculated from the beam forming in the numerical simulations (Fig. 1c) and this value matches Snell’s law prediction *θ* = arc cos(*v*_*s*_/*v*_*R*_) with *v*_*R*_ being the Rayleigh wavespeed (see supplementary video material). The observed turning point in both cases, the classic and inverse wedges, remains approximately the same.

## Discussion

**Figure 3:**
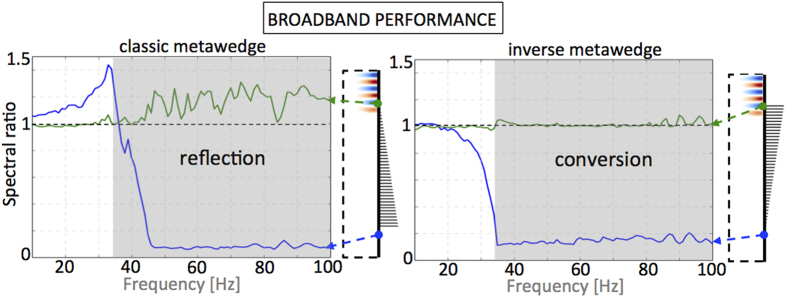
The efficiency of the metawedge shown via the spectral ratio (i.e. spectral density of the displacement in the substrate with the wedge divided by the spectral density measured in the bare halfspace) calculated just before (green) and just after (blue) the wedge. The sharp reduction, over a very broad range of frequencies, is indicative of the protection afforded by the metawedge.

**Figure 4:**
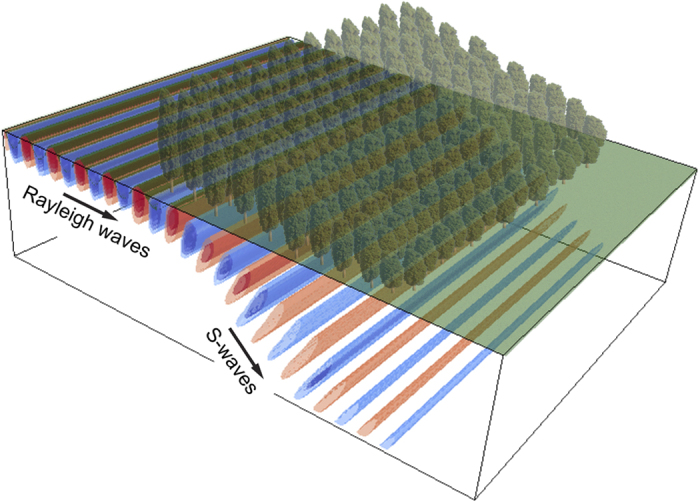
Artist view of the conversion phenomena allowed by the metawedge rendered from the 2D simulation results. An experiment could be potentially realised in a forest where trees are arranged with decreasing height with respect to the Rayleigh wave source.

In summary we have shown that a graded vertical array of resonators arranged atop an elastic substrate can act in two distinct regimes: one, the classical wedge, that traps Rayleigh waves and provides an effect akin to the rainbow effect^8^ and the other, the inverse wedge, generates mode conversion with surface waves diverted into the bulk. In both cases, it is striking that this resonant metawedge has a considerable broadband range of protected frequencies, as shown in Fig. 3, and this is strongly indicative that this mechanical metamaterial will have application for protection from vibration, and in elastic wave filtering, and given the ubiquity of elastic waves in a range of applications from surface acoustic wave devices through to seismology and civil engineering. As recently demonstrated in^7^, the dynamic properties and dimensions of the vertical resonators used in this study closely resemble those of forest trees (an example of natural metamaterials). Therefore a fascinating experiment at the geophysical scale can be designed using trees of decreasing height randomly arranged to form a wedge-like profile as represented by the artwork in Fig. 4.

## Methods

The propagation of seismic waves in a 2D halfspace is a well-known problem in numerical seismology and modeled by solving the *P* − *SV* elastic wave equation (*P*: Primary or compressional wave, *SV*: vertically polarized shear or Secondary wave), but its coupling with resonating tree-like elements is rather unusual. The accuracy of the method has been thoroughly tested using plate and rods as input model and it has delivered excellent results. The 2D time domain simulations are carried out using SPECFEM2D a code that solves the elastic wave equation using finite difference in time and the spectral element method in space. The parallelization is implemented through domain decomposition with MPI. The mesh is made of quadrilateral elements and it is generated using the commercial software CUBIT. The top surface is traction free to support the propagation of surface waves, the other boundaries are supplied with perfectly matched layer conditions and are labelled as PML in Fig. 1. Simulations are then run on a parallel cluster (Froggy at University of Grenoble) on 16 CPUs. 2D and 3D plots have been generated with Paraview.

## Additional Information

**How to cite this article**: Colombi, A. *et al*. A seismic metamaterial: The resonant metawedge. *Sci. Rep.* **6**, 27717; doi: 10.1038/srep27717 (2016).

## Supplementary Material

Supplementary Information

Supplementary Information
