# Supplementary material for: A seismic metamaterial: The resonant metawedge
Source: Sci Rep. 2016 Jun 10;6:27717. doi: 10.1038/srep27717 (PMC4901369; doi:10.1038/srep27717)
Supplement: Supplementary Information [file srep27717-s1.pdf]

Supplementary Materials for
A seismic metamaterial: The resonant metawedge

Andrea Colombi*, Daniel J. Colquitt, Philippe Roux,
Sebastien Guenneau, Richard V. Craster

*Corresponding author. Email: andree.colombi@gmail.com

Here we derive the dispersion equation governing the propagation of surface waves through the array of resonators. We also provide a closed form expression for the frequency at which the hybridisation and conversion of surface waves to bulk waves occurs.

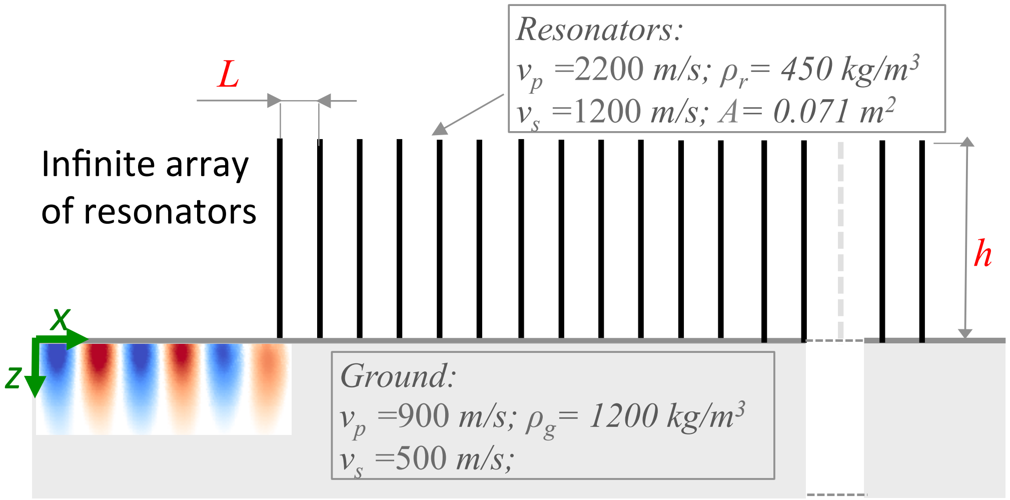

Figure 1: 2D model of the array of resonators connected to the halfspace.

The metawedge presented in the main text can be reduced to a two-dimensional problem for plane surface waves travelling parallel to the x-axis (see figure 1). In particular, we consider the problem of a doubly-periodic array of slender parallel elastic resonators resting on the surface of an elastic half-space, occupying the region $z < 0$ (Fig. 1). The resonators are of height h, cross-sectional area A, have Young's modulus E_r, and density ρ_r, and are periodically distributed over the plane $z = 0$. The elementary cell is denoted by $\Omega = \{\boldsymbol{x} : -L/2 < x < L/2, -L/2 < y < L/2, z \leq 0\}$, with $\boldsymbol{x} = (x, y, z)$ being the spatial variables. The resonators are defined by the set $\mathcal{R} = \{\boldsymbol{x} : x = 0, y = 0, 0 \leq z < h\}$. The three-dimensional vector displacement amplitude field $\boldsymbol{u} = \boldsymbol{u}(\boldsymbol{x})$ is governed by the following boundary value problem

$$\nabla \cdot \mathbf{C} : \nabla \boldsymbol{u} + \rho_b \omega^2 \boldsymbol{u} = \mathbf{0} \quad \text{for} \quad \boldsymbol{x} \in \Omega, \tag{1a}$$

$$\left(\frac{\partial^2}{\partial z^2} + \frac{\omega^2}{v_r^2} \right) u_2(z) = 0 \quad \text{for} \quad \boldsymbol{x} \in \mathcal{R}, \tag{1b}$$

$$(\mathbf{C} : \nabla \boldsymbol{u}) \cdot \boldsymbol{n} - [V\delta(x)\delta(y), 0]^{\mathrm{T}} = \mathbf{0} \quad \text{for} \quad \boldsymbol{x} \in \Omega \cup \mathcal{R}, \tag{1c}$$

where $\mathbf{C}$ and ρ_b are the usual elastic stiffness tensor and density corresponding to the half-space, ω is the angular wave frequency, v_r is the compressional wave-speed in the resonators, and $V = A\omega\sqrt{E_r\rho_r}\tan(h\omega\sqrt{\rho_r/E_r})u_3(\mathbf{0})$ is the vertical force exerted on the half-space by the resonators. Bloch-Floquet quasi-periodicity conditions are also imposed on the boundary of the elemetnary cell.

In physical terms, equation (1a) is the equation of motion governing the propagation of monochromatic waves of angular frequency ω in an elastic halfspace (i.e. the ground); equation (1b) is the equation of motion governing the propagation of time-harmonic compressional waves in a thin elastic rod (i.e. the resonators); finally, equation (1c) represents the continuity of tractions (that is, the balance of forces from Newton's 3rd law of motion) across the junction between the resonators and the halfspace.

The displacement field permits the usual Helmholtz decomposition $\boldsymbol{u} = \nabla\phi + \nabla\times\boldsymbol{\psi}$, where both potentials satisfy the Helmholtz equation (see *(1, 2)*, among others)

$$\nabla^2\phi + \frac{\omega^2}{v_p^2}\phi = 0, \quad \nabla^2\boldsymbol{\psi} + \frac{\omega^2}{v_s^2}\boldsymbol{\psi} = \mathbf{0}, \quad \text{for} \quad \boldsymbol{x}\in\Omega \tag{2}$$

and v_p and v_s are the compressional and shear wave-speeds of the elastic bulk respectively. The shear potential vector must also satisfy the incompressibility condition $\nabla\cdot\boldsymbol{\psi} = 0$.

For the the deep sub-wavelength regime of interest in the current configuration, it is sufficient to search for solutions of the form

$$\phi(\boldsymbol{x}) = \phi_0 e^{ikx+\alpha z}, \quad \alpha = \sqrt{k^2 - \omega^2/v_p^2}, \tag{3a}$$

$$\boldsymbol{\psi}(\boldsymbol{x}) = \boldsymbol{\psi}_0 e^{ikx+\beta z}, \quad \beta = \sqrt{k^2 - \omega^2/v_s^2}, \tag{3b}$$

where the branches are chosen such that $\Re\alpha > 0$ and $\Re\beta > 0$, thus ensuring decay as $z\to-\infty$. The two Ansätze (3) correspond to monochromatic plane waves that propagate with constant amplitude along the x-axis and decay, exponentially, into the bulk. Substituting these Ansätze into the surface tractions conditions (1c) and integrating over $(x, y)\in(-L/2, L/2)^2$ yields

$$4\xi^2\sqrt{\xi^2 - r^2}\sqrt{\xi^2 - 1} - (2\xi^2 - 1)^2 = \sqrt{\xi^2 - r^2}\tan(h\omega\sqrt{\rho_r/E_r})\frac{A}{L^2}\sqrt{\frac{E_r\rho_r}{\mu_b\rho_b}}, \tag{4}$$

where $\xi^2 = k^2v_s^2/\omega^2$, μ_b is the bulk shear modulus, and $r^2 = v_s^2/v_p^2$. Equation (4) is the dispersion equation for plane surface waves propagating through the array of resonators parallel to the x-axis (or equivalently, the y-axis). Although (4) does not have closed form solutions, it can be expressed as a sixth-order polynomial in ξ^2 allowing the roots to be computed efficiently. The coefficients of the sextic polynomial are rather unwieldy and, in the interests of brevity, are not provided here.

The intersection of the dispersion curves with the shear-wave light-line is associated with the frequency at which hybridisation and the mode-conversion of surface to bulk waves occurs.

Setting $\xi = kv_s/\omega = 1$ in (4), we obtain a convenient expression for the frequencies at which hybridisation occurs

$$f = \frac{v_r}{h}\left[\frac{n}{2} - \frac{1}{2\pi}\arctan\left(\frac{L^2}{A\sqrt{1-r^2}}\sqrt{\frac{\mu_b\rho_b}{E_r\rho_r}}\right)\right], \quad \text{where} \quad n = 1, 2, 3, \ldots. \tag{5}$$

A video representing the conversion of Rayleigh into shear waves is provided as supplementary material. The colorcode represents the vertical component of the displacement. Results are given for two different frequencies. Notice that the angle θ remains exactly the same.

References and Notes

1. J. W. Gibbs, E. B. Wilson, *Vector calculus* (Yale University Press, 1929).

2. G. B. Arfken, *Mathematical methods for physicists* (Academic press, 2013).
